# Expression of Concern: Barriers to cleaning of shared latrines in slums of Addis Ababa, Ethiopia

**DOI:** 10.1371/journal.pone.0329218

**Published:** 2025-07-29

**Authors:** The PLOS One Editors

The *PLOS One* Editors issue this Expression of Concern to alert readers that this article [1] was identified as one of a series of submissions for which we have concerns about authorship and adherence to the journal’s first and second publication criteria. In addition, the article’s ethics statement does not report an ethics approval number or date; this issue was not resolved in follow-up discussions with the authors.

We regret that these issues were not addressed prior to the article’s publication.
