# Supplementary material for: Barriers to cleaning of shared latrines in slums of Addis Ababa, Ethiopia
Source: PLoS One. 2022 Mar 10;17(3):e0263363. doi: 10.1371/journal.pone.0263363 (PMC8912180; doi:10.1371/journal.pone.0263363)
Supplement: S1 Questionnaire — (DOCX) [file pone.0263363.s001.docx]

| **Part I :- Socio-economic and demographic factors** | | |
| --- | --- | --- |
| No. | Questions | Response option Code of response |
| 1 | Who is the head of the household? | Father 1  Mother 2  Other/specify __________ |
| 2 | Age of the study participant [incomplete years]? | __________year |
| 3 | Sex of the study participant | Male 1  Female 2 |
| 4 | occupation type of the study participant | Housewife 1  Daily laborer 2  Government employee 3  Merchant 4  Private investor [company] employee] 5  Other/specify __________ |
| 5 | Education status of the study participant? | Illiterate 1  literate informal 2  literate formal [mention highest grade completed] 3 _______ |
| 6 | Marital status of the of the study participant? | Married 1  Single 2  Windowed 3  Divorced 4 |
| 7 | Average household monthly income [from all sources]? | _______In birr |
| 8 | Total family size of the household?  Number of households shard the latrines of one door only | _______In number    ------------------------------  -------------------------------- |
| 9 | Ownership status of the living house? | Rented from government 1  Rented from private 2  Owned 3  Neither rented nor owned 4 |
| 10 | How many rooms does the house have? | ________ In number |

11. Superstructure material of the public latrine

A. Iron sheet/mud/wood C. Floor/slab material

B. Bricks/stone D. Mud/wood

E. Stone /slab

12. Did you feel privacy during using public latrine?

1. Yes
2. No

13. Does the public latrine have a door that you can able to close during defecation?

1. Yes
2. No

14. Does the door hold in place?

1. Yes
2. No

15. Does it have a locking latch?

1. Yes
2. No

16. Does the public latrine have a complete superstructure?

1. Yes
2. No

17. Does the superstructure offer privacy?

1. Yes
2. No
3. Does the public latrine have a roof that able to prevent rain flow inside the latrine?
4. Yes
5. No
6. Does the public latrine have a locked door or do you have key for locked and open?
7. Yes
8. No
9. Does the facility offer good ventilation?
10. Yes
11. No
12. Does the facility have a good wall that able to prevent someone seeing you during defection?
13. Yes
14. No

**The slab and other visible factors**

1. Are there cracks/visible spaces on the slab?
2. Yes
3. No
4. Is the drop hole too big?
5. Yes
6. No
7. Is the drop hole open?
8. Yes
9. No
10. Are there standing fluids on the slab?
11. Is the facility full?
12. Yes
13. No
14. Is the facility semi-full?
15. Yes
16. No

1. Do the users have principles in shared sanitation?
2. Yes
3. No

If your answer is yes explain

29. Does Users participate collectively in decisions?

1. Yes
2. No

30. Does Users experience conflict?

1. Yes
2. No

31. Does they have Conflict resolution mechanisms?

1. Yes
2. No

32. Do they have mechanism on Monitoring of the toilet and users?

1. Yes
2. No

If your answer is yes explain

33. Is there any rules of use?

1. Yes
2. No

If your answer is yes explain

34. Do you think the toilet you use is clean or improved?

1. Yes
2. No

If your answer yes why?

If your answer No why?

35. Does quality of toilet affect our health?

1. Yes
2. No

If your answer is yes explain

If your answer is no explain

36. Is there enough water supplies for cleaning?

1. Yes
2. No

37. What are most common effects of using unsafe toilets?

38. Reason for not cleaning the toilets?

39. What is your understanding on a clean toilet?

1. No faeces
2. Toilet does not smell
3. Toilet room has no flies
4. Floor soaked with urine
5. Faeces on toilet walls
6. Toilet un ventilated

40. What are you using for cleaning the toilet?

|  | **Hygiene related** |  |
| --- | --- | --- |
| 41 | Do you have hand washing facilities [container] near the latrine? [Observe] | No 0  Yes 1 |
| 42 | Is there water in the latrine hand washing facility [container]? [Observe] | No 0  Yes 1 |
| 43  44 | Is there soap near the latrine hand washing facility? [Observe]  Cleanness of the toilet (observe) | No 0  Yes 1 |

Qualitative interview check list for respondent and key informant interview

45. Any opinion about using sharing of latrines

46. about security and dignity concerns of using shard public latrines

47. Any challenges at night of shared latrine users?

48. Any challenges at day time of shared latrine users?

49. Any challenges for the government for keeping shared latrine clean?
