# Supplementary material for: Barriers to cleaning of shared latrines in slums of Addis Ababa, Ethiopia
Source: PLoS One. 2022 Mar 10;17(3):e0263363. doi: 10.1371/journal.pone.0263363 (PMC8912180; doi:10.1371/journal.pone.0263363)
Supplement: S2 Questionnaire — (DOCX) [file pone.0263363.s002.docx]

**ክፍል 1: - ማህበረ ኢኮኖሚያዊ እና የህዝባዊ መረጃዎች**
1. የቤተሰቡ ራስ ወይም ባለቤት ማን ነው?

አባት 1
እናት 2
ሌላ / በትክክል ይግለጹ __________
2. የጥናቱ ተሳታፊ እድሜ ዓመት? __________አመት
3. የተሳታፊ ፆታ

ወንድ 1
ሴት 2
4. የጥናቱ ተሳታፊ ሃይማኖት?

ክርስቲያን 1
ሙስሊም 2
ሌላ / በትክክል ይግለጹ __________

5. የተሳታፊው የትምህርት?
የቤት እመቤት 1
የቀን ሰራተኛ 2
የመንግስት ሰራተኛ 3
ነጋዴ 4
የግል ኢንቨስትመንት [ኩባንያ] ተቀጣሪ ሠራተኛ 5
ሌላ / በትክክል ይግለጹ __________

6. የጥናቱ ተሳታፊ የትምህርት ሁኔታ?

ማንበብና መጻፍ 1
ማንበብና መጻፍ 2
መማርያነት [በይፋ የተጠናቀቀው ደረጃ ተጠናቅቋል] 3 _______

7. የተሳታፊ የትዳር ሁኔታ?

ያገባ 1
ነጠላ 2
በሞት የተለዩ 3
ተፋቷል 4
8. አማካኝ የቤተሰብ ገቢ [ከሁሉም ምንጮች]? _______ ብር
9. ጠቅላላ የቤተሰብ ቤተሰብ ብዛት?
10. የአንድ ቤት በር ብቻ የሕዝብ መጸዳጃ ቤቶችን ተጠቃሚ
11. የጋራ መፀዳጃ ቤቱን የሚጠቀሙ ሰዎች ብዛት ------------------------------
12. የቤቱ ባለቤት የባለቤትነት ሁኔታ?

ከመንግስት ተከራይ 1
ከግል ተከራይቷል 2
ባለቤትነት 3
አልከራዩም ወይም አልተያዙም 4
13. ቤቱ ምን ያህል ክፍሎች አሉት? ________ በቁጥር

14. የሕዝብ መጸዳጃ ቤቶች በክረምት ወራት ጊዜ ሞልተው ይሞላሉ ወይ?
ሀ. አዎ
ለ. አይ
15. ትርፍ ፍሰት ወደ ሌሎች ቤቶች ይገቡ ይሆን?
ሀ. አዎ
ለ. አይ
16 . የህዝብ መጸዳጃ ቤት እቃዎች / ቁሳቁሶች /
ሀ. የብረት እጀታ / ጭቃ / እንጨት C. ወለል / ስሌት
ለ. ጡቦች / ድንጋይ መ.ስለ / እንጨት   ሠ. ድንጋይ / ሳጥ
17. የሕዝብ መጸዳጃ ቤት በሚጠቀሙበት ጊዜ የግል ምቾቶችሁ የተጠበቁ ናቸው?
ሀ) አዎ
ለ) የለም
18. የሕዝብ መጸዳጃ ቤት መጸዳጃ ቤትን መዝጋት የሚችሉት በር ነውን?
ሀ) አዎ
ለ) የለም
19. በሩ እላዩ ልይተያይዟል?
ሀ) አዎ
ለ) የለም

20. መቆለፊያ ቦርቻ አለው?
ሀ) አዎ
ለ) የለም
21. የሕዝብ መፀዳጃ ቤት የተሟላ አሠራር አለው?
ሀ) አዎ
ለ) የለም

22. የመፀዳጃ ቤቱ አሰራር ለግላዊነት መጠቀሚያ ይሰራል?
ሀ) አዎ
ለ) የለም
23. የሕዝብ መፀዳጃ ቤት በዝናብ ወቅት ውስጥ የውኃ ፍሰትን ለመከላከል የሚችል ጣራ አለ ወይ?
ሀ) አዎ
ለ) የለም

24. የሕዝብ መጸዳጃ ቤት መቆለፊያ በር አለው ወይ ወይስ ለቁልፍ እና ለመክፈት ቁልፍ አለው?
ሀ) አዎ
ለ) የለም

25. መፀዳጃ ቤቱ በቂ አየር ያገኛል?
ሀ) አዎ
ለ) የለም

26. መፀዳጃ ቤቱ በተያዘበት ወቅት ሌላ ሰው እንዳያገኝዎት የሚያስችል ጥሩ ግድግዳ አለው?
ሀ) አዎ
ለ) የለም
**የታችኛው እና ሌሎች ተጨባጭ ሁኔታዎች**
27. በእንጣፉ ላይ ጥንብሮች / የሚታዩ ቦታዎች አሉ?
ሀ) አዎ
ለ) የለም

28. የመፀዳጃው ጉድጉድ ትልቅ ነው?
ሀ) አዎ
ለ) የለም

29. በመፀዳጃ ቤቱ ላይ ፍሳሽ ይታያል?
ሀ) አዎ
ለ) የለም

30. መገልገያው ሙሉ ነው?
ሀ) አዎ
ለ) የለም

31. ተቋሙ በከፊል የተሞላ ነው?
ሀ) አዎ
ለ) የለም
32. ተጠቃሚዎች በጋራ የንጽህና መርሆዎች አሉት?
ሀ) አዎ
ለ) የለም
 መልስዎ አዎ ከሆነ, ያብራሩ

33. ተጠቃሚዎች በአጠቃሊይ በዉሳኔዎች ይሳተፋሉ?
ሀ) አዎ
ለ) የለም

34. ተጠቃሚዎች እርስ በርስ ይጋጫለ?
ሀ) አዎ
ለ) የለም

35. የግጭት አፈታት ስልቶች አሉን?
ሀ) አዎ
ለ) የለም
36. የመፀዳጃ ቤቶችን እና ተጠቃሚዎችን ክትትል በተመለከተ አግባብ አላቸውን?
ሀ) አዎ
ለ) የለም
መልስዎ አዎ ከሆነ, ያብራሩ

37 . የአጠቃቀም መመሪያ አለ?
ሀ) አዎ
ለ) የለም
መልስዎ አዎ ከሆነ, ያብራሩ

38. የመፀዳጃ መጠቀሚያዎች ንጹህ ወይም የተሻሻለ ይመስልዎታል?
ሀ) አዎ
ለ) የለም
   የእርስዎ መልሶች አዎ ካሉ ለምን?

  መልሱ አይደለም ከሆነ ለምን?

39. የመፀዳጃ ቤቱ ጥራት በጤናችን ላይ ተጽዕኖ ያሳድራል?
ሀ) አዎ
ለ) የለም
መልስዎ አዎ ከሆነ, ያብራሩ

መልስዎ አይደለም ከሆነ ማብራሪያ

40. ለመጸዳጃ የሚሆን በቂ ውሃ አለ?

ሐ) አዎ
ለ) አይ

41. በንጹህ መፀዳጃ ቤት ላይ ምን አይነት ነገር አለ?
ሀ. ምንም ፍሳሽ የለም
ለ. የሽንት ቤት ምንም አይነት ሽታ የሌለው
ሐ. የሽንት ቤት ክፍል ምንም ዝንብ የለውም
መ. በሽንት የተሸፈነ ወለል
ሠ. በመጸዳጃ ግድግዳዎች ላይ ያሉ ሰገራዎች
ረ. ነፋሻማ ያልሆነ ሽንት ቤት

42 . ንጹህ ያልሆኑ መጸዳጃዎችን መጠቀም አብዛኛው የተለመደው ውጤት ምንድነው?

43. መጸዳጃ ቤቱን ያለማጽዳት ምክንያቶች?

44 . የመፀዳጃ ቤት ለማፅዳት ምን ትጠቀማላችሁ?

**የግል ንጽህናን በተመለከተ**
45. በሽንት ቤት አቅራቢያ የእጅ መታጠቢያ እቃዎችን አሉ? [አስተውሉ]

የለም 0
አዎ 1

46. በእጅ መታጠቢእቃ ውስጥ መታጠቢያ ውኃ አለ? [አስተውሉ]

የለም 0
አዎ 1

47. ከመፀዳጃ ቤት አጠገብ እጅ መታጠቢያ አጠገብ ያለው ሳሙና አለ? [አስተውሉ]

የለም 0
አዎ 1

**ለጥያቄ መልስ እና ለቃለ መጠይቅ / ቃለ-ምልልስ ቃለ መጠይቅ የጥራት ቃለ-መጠይቅ ዝርዝር
የህዝብ መፀዳጃዎችን ስለመጠቀም አስተያየት**

48. የሕዝብ መጸዳጃ ቤቶች ስለመጠቀም እና ስለ ክብር እና የደህንነት ስጋቶች


49. ህዝባዊ የመፀዳጃ ቤት ተጠቃሚዎች በምሽት ያጋጥማቸውን ችግሮች በተመለከተ?


50.በሕዝብ መጸዳጃ ቤት ውስጥ ለቀን ተጠቃሚዎች ያጋጠሙ ተግዳሮቶች?


51. የመንግሥት መፀዳጃ ቤት ንጽሕና እንዲጠበቅ መንግሥት የሚያጋጥሙ ችግሮች አሉ?
