# Supplementary material for: Barriers to cleaning of shared latrines in slums of Addis Ababa, Ethiopia
Source: PLoS One. 2022 Mar 10;17(3):e0263363. doi: 10.1371/journal.pone.0263363 (PMC8912180; doi:10.1371/journal.pone.0263363)
Supplement: Attachment — Submitted filename: Manuscript with track changes.docx [file pone.0263363.s004.docx]

**Barriers to Cleaning of Shared Latrines in Addis Ababa Slums: Unconditional Logistic Regression Analysis**

Kidist Hailu^1#^, Metadel Adane^2*#^

^1^Bethzatha General Hospital, Addis Ababa, Ethiopia

^2^Department of Environmental Health, College of Medicine and Health Sciences, Wollo University, Dessie, Ethiopia

*E-mail: [metadel.adane2@gmail.com](about:blank)

^#^Contributed equally

# Abstract

Shared latrines and other shared sanitation facilities are vital for communities that lack private latrines. However, the cleanliness of shared latrines continues to be a problem in sub-Saharan Africa, including slums of Addis Ababa, Ethiopia. Investigating the barriers to keeping shared latrines cleaning may inform the comprehensive sanitation programs in slums of Addis Ababa. Therefore, this study was designed to examine the barriers to cleaning of shared latrines in slums of Addis Ababa, Ethiopia. A community-based unmatched case-control study was conducted between September and November 2017 in a slum district in Addis Ababa. Cases were defined as users of shared latrines that had reported not cleaning the shared latrine during the week prior to data collection, whereas controls were shared latrine users who had reported cleaning the shared latrine during the week prior to data collection. Data were collected using a structured questionnaire and direct observation checklist, and were entered, cleaned and analyzed using Statistical Package for the Social Sciences (SPSS) version 23.0. Unconditional logistic regression models were used for data analysis. Bivariate (crude odds ratio [COD]) and multivariable (adjusted odds ratio [AOR]) unconditional logistic regression analyses were employed. From the adjusted analysis, variables with *p*<0.05 were considered as statistically significant barriers to cleaning of shared latrines. Majority of controls 174(87.0%) and about one-third 31(31.0%) cases considered the shared latrines they used to be unclean or in need of repair. About half 99(49.5%) of shared latrines used by cases and almost one-third 32(32%) of the shared latrines used by controls had visible cracks and spaces in the floor and slabs. The barriers to cleaning of public latrines were found to be monthly household income less than $55.60 USD (AOR = 1.8; 95%CI: 1.2-3.10), users feeling a lack of privacy during latrine use (AOR = 2.95; 95% CI: 1.60 - 5.43), no locking latch on the latrine door (AOR = 4.60; 95% CI: 2.43 - 8.79), inadequate ventilation of latrine (AOR: 4.88; 95% CI: 2.44 - 9.63), lack of regular monitoring of latrine by health extension workers (AOR = 2.86; 95%CI: 1.32 - 6.21) and a lack of enough water at home for cleaning the latrine (AOR = 4.91; 95% CI: 1.07 - 9.48). The major barriers to cleaning of public latrines in slums of Addis Ababa were monthly household income, users feeling lack of privacy during latrine use, no locking latch on the latrine door, adequate ventilation of latrine, shared latrine not regularly monitored by health extension workers and lack of enough water at home for cleaning the latrine. We recommended promoting cleaning of shared latrines by designing programs to increase household income, to improve latrine privacy by modifying superstructure and including a door with locking latch to make adjust for better ventilation, to ensure regular monitoring of latrines by health extension workers and to make enough water available for latrine regular latrine cleaning.

# Background

Rapid expansion of urbanization in developing countries resulted the creation of urban slums [1, 2], which is characterized by one or more of the features of overcrowding, inadequate safe water supply, insecure property tenure, inadequate drainage and sewage networks, and lack of sanitation or proper solid waste disposal [2-6]. Shared latrines, including public latrines, are common in African slums while a lack of space for construction of private latrines is a big challenge [1, 5, 7]. Shared facilities can reduce stress when proper maintenance and management systems are in place [8]. However, slums in sub-Saharan Africa experience elevated disease risks due to poor sanitation infrastructure that does not reliably sequester excreta to prevent human exposure [9, 10].

A study in Indonesia and Bangladesh showed that an increased number of users appeared to negatively affect toilet cleanliness and that when sanitation facilities were clean and shared by a limited number of households, users of shared latrines often reported feeling both satisfied and safe [11]. During the rainy season, unsealed shared latrines in slums may overflow and disperse pathogens [12] and diarrhea incidence in slums tends to peak [13], resulting in higher diarrhea transmission among children under five than during the dry seasons. Furthermore, lack of cleaning of shared latrines in slums worsens the already bad sanitation situation [14].

Heijenen *et al.* reported that shared latrines were generally in poorer condition than those latrines that were not shared [15, 16]. The number of users per shared sanitation facility tends to be inversely related to the cleanliness of the facility [17, 18]. A shared latrine can be equated with a common good whose management depends on the users. If users do not work collectively towards keeping the facilities clean, their quality and utilization may decline [19].

In Africa’s urban slums, where private latrines are lacking, there is a great demand for public latrines. Therefore, improving their hygienic status is vital for minimizing disease outbreaks in these areas [3]. Bartlett (2003) found that the lack of latrines in poor communities caused many people to defecate in the open or into plastic bags and papers that were then discarded with the household garbage [20]. A recent study in eastern Ethiopia found a lack of effective social mobilization to be the main cause for open-defecation [21].

Disposal of garbage close to homes was a significant risk factor for high fly densities and the presence of flies around the shared latrines [22]. Strina *et al*. found that people in latrine-owning households in Salvador, Brazil behaved more hygienically than those without latrines [23]. A similar study in Rajshahi City slums in Bangladesh revealed that feces were observed around 61.0% of the latrines [24]. Unsanitary conditions of latrines and poor hygiene behavior were related to increased diarrhea episodes in slums of An-Nasr in Jordan, Tebbaneh in Libya [25] and Ikare-Akoko in Nigeria [26]. Findings that substandard latrine construction contributes to the presence of flies indicate that improved superstructures may decrease fly densities around latrines [27, 28].

Improving cleaning practices of shared latrines is a step toward achieving the United Nations’ 2030 goals for Sustainable Development, in line with achieving Target 6.2 of the Sustainable Development Goal (SDG) of universal access to sanitation as a key priority [29]. However, evidence in urban areas has indicated that shared latrines are often poorly maintained, which discourages their regular use and potentially increase disease risk [30].

In Ethiopia, Addis Ababa is an example of a city characterized by informal, overcrowded settlements that have shared latrines that are in poor hygienic conditions [31, 32]. This situation increases the risk of diarrheal diseases, respiratory infections and other diseases [33, 34]. For example, in 2008, the city-wide data on basic indicators in Addis Ababa reported that 26% of the houses and the majority of slum dwellers had no toilet facility, 33.0% of households shared a latrine among more than six households and 71.0% of the households did not have adequate sanitation [31]. Furthermore, studies in slums of Addis Ababa indicated that poorly maintained shared latrines may have contributed to contamination of household water supplies as a result of the proximity of many latrines and crowded neighborhoods [34, 35].

The Joint Monitoring Program (JMP) excludes what it calls ‘shared’ sanitation facilities-those used by two or more households-from the definition of ‘improved sanitation’ due to the concern that shared toilets tend to be less hygienic than private ones [36]. However, there is limited existing information on why shared latrines are not cleaned by users in slum contexts. A previous study done in slums of Addis Ababa found that sanitation and hygienic levels of shared latrines were low, although the main reasons for this could not be addressed [34]. To address this gap in knowledge, this study examines barriers to cleaning of shared latrines in slums of Addis Ababa, Ethiopia.

# Methods

## Description of the study area

The study was conducted in District 05 in Lideta Sub-City (Fig. 1). Addis Ababa has 116 districts (the lowest administrative unit in Addis Ababa). In the absence of a clear demarcation of the slums in Addis Ababa, the UN-HABIAT study in 2010 estimated that 80% of Addis Ababa was slums, where most of the residents lived in houses rented from the local government [32]. A previous study in slums of Addis Ababa reported that 51.0% of slum residents used public latrines (shared by two or more households) and 26.8% of them also used pit latrines that were without a slab floor [34].

Fig 1. Map of the study area

## **Study design, source populations, inclusion and exclusion criteria**

A community-based unmatched case-control study was conducted between September and November, 2017. Cases were users of shared latrines who by self-report had not cleaned the latrine during the week prior to data collection, whereas controls were users of shared latrines who by self-report had cleaned the latrine during the week prior to data collection. The source population was all dwellers in slums of Addis Ababa who had used shared latrines during the week prior to data collection, whereas the study population was District 05 residents in Lideta Sub-City who had used shared latrines during the week prior to data collection. In this study, latrine users that are responsible for the cleaning of their latrines were included, whereas latrine users that not are responsible for the cleaning of their latrines were excluded. Households that had private latrines and did not share with others were excluded.

## **Sample size determination**

The sample size was determined using Epi Info version 7.0 with the consideration of proportion (p) of households that had not cleaned a shared latrine within one week prior to data collection was taken as 50.0% due to lack of studies in a similar setting, and expected odds ratio of 1.5 used based on another study suggestion to obtain adequate sample size [37]. Furthermore, power of 80.0% and control-to-case ratio of 2:1 was also used in line with the Pitman Efficiency assumption [38]. Considering a 10% non-response rate, the final adequate sample size becomes 100 cases and 200 controls, for a total of 300 households of users of shared latrines.

## **Sampling procedures**

Through house-to-house visits, cases were selected from those who had not cleaned the shared latrine at least once during the week prior to data collection and controls were selected from shared latrine users who had cleaned the latrine at least once during the week prior to data collection. Study participants who were not available during the survey were revisited once on the same day or the next day. If not available again, the study participant was considered a non-respondent. Since most of the slum dwellers were day laborers and the study area was business areas, the data collection was performed Saturdays at the mid-day and Sunday afternoons every weekend to enable individuals be more likely to be home to participate in the survey. However, since most commonly, slum dwellers are at home on Sunday afternoons, we considered this cultural norm to make all study participants easily accessible for interviews.

## **Operational definition**s

**Shared latrines**: Refers to latrines shared by two or more households, including public latrines that are used by known households with a shared responsibility of cleaning by the users, whereas public latrines publicly accessible for everyone at a market, school and main road were excluded from this study.

**Shared latrine use**: Individuals included in both the case and control groups might use the same or different latrines; this study investigated barriers to latrine cleaning during the week prior to data collection.

**Latrine access**: Means that individuals only accessed the latrines that were within a certain distance from their homes as provided by *kebeles* for shared use, or use of their own shared latrine, or use of a latrine that is part of a household and also shared by two or more households.

**Barriers to cleaning of shared latrines**: Obstacles that prevented users from cleaning shared latrines.

**Unmatched case-control study**: Matching variable at the design stage was not used during selection of cases and controls study participants.

**Cases**: Users of shared latrines who self-reported that they had not cleaned the latrine during the week prior to data collection.

**Controls**: Users of shared latrines who self-reported that they had cleaned the latrine during the week prior to data collection.

**Unconditional logistic regression**: A model used for data analysis of unmatched case-control studies, where matching for case and control is not performed at the design stage [39].

**Slums**: Urban areas dominated by informal settlement (by illegal settlers) that are characterized by one or more of the five slum characteristics (overcrowding, poor sanitation, insecure land tenure, lack of access to water supply, poor housing quality and other infrastructure) [2].

## **Data collection and data quality assurance**

Household survey data was collected using a pretested questionnaire and an observational checklist. The questionnaire and the observational checklist were first prepared in English and then translated into (local language) Amharic for participating household use. Three data collectors who were BSc in environmental health professionals administered the survey by interviewing the study households who were 18 years old or older and also by direct observation of the shared latrine. Data collectors were trained by the principal investigator for two days on the aim of the study, the content of the questionnaire and observation checklist, ethical issues and approaches during data collection.

During data collection, study participants were asked to show their latrine for the purpose of recording the observational data regarding superstructure of the latrine, latrine door and locking latch, slab, fullness of latrine, handwashing facilities, availability of water and soap. Daily supervision was carried out by one supervisor and principal investigator to check the completeness of the questionnaires and consistency of the data. The completeness of questionnaires was checked daily before data entry.

To ensure the quality of the data, we also pre-tested the questionnaire among 10 cases and 20 control households (10% of the sample size) in one non-selected area of District 4 in Lideta Sub-City to evaluate its face validity and to ensure that the participants understood the questions. Any amendment made to the questionnaire such as avoiding of unnecessary questions, confusing term usage, and adding of important questions was based on the pre-test. Inter-observer reliability was ensured by providing clear definitions about shared latrines, cases and controls, latrine cleaning, superstructure of the latrine and events to be recorded by training data collectors, and by providing feedback about discrepancies during daily supervision.

Also, 10% of the study participants were re-interviewed by another interviewer to check reliability of the information collected by different interviewers. The qualifications of the interviewers and the training they received also reduced the likelihood that interviewer bias occurred. Entered data was re-entered on 10% of the sample to check the consistency or reduce data entry error. After the data was entered and cleaned, quality assurance measures were employed using descriptive statistics from cross-tabulation and frequency distribution before performing statistical analysis.

## **Data management and analysis**

The collected data were coded and entered in to EpiData version 3.1 and exported to Statistical Package for the Social Sciences (SPSS) version 23.0 for cleaning and analysis. Descriptive statistics were carried out, including means ±SD (standard deviations) for continuous variables. The presence of multi-collinearity among independent variables was checked using standard error at the cutoff value of 2 and we found there was no multi-collinearity between independent variables with a maximum standard error of 1.67.

Unconditional logistic regression model was used for data analysis. The modeling strategy involved estimating the bivariate analysis (crude odds ratio [COR]) and multivariable analysis (adjusted odds ratio [(AOR)] at 95% CI. From the multivariable analysis, the barriers to cleaning of public latrines were examined by controlling potential confounders using the methods explained elsewhere [40]. Hosmer-Lemeshow statistic was used to test the goodness-of-fit of the model. The adjusted model estimated the overall effect of all variables to select the significant barriers after adjustment for confounding factors. From the adjusted analysis, variables with *p* < 0.05 were taken as statistically significant and independently associated with barriers to cleaning of shared latrines in slums of Addis Ababa.

## Ethical considerations

Ethical clearance was obtained from GAMBY College of Medical and Business Institutional Review Board (IRB). Permission to undertake this study was also obtained from Addis Ababa Health Bureau, slum District 05 in Lideta Sub-City. Written consent was obtained during recruitment of the study participants. During observation of shared latrines, study participants who were using unclean latrines were advised to keep their latrine in a hygienic condition. Study participants also were informed that participation was affirmed by the procedure of probability sampling technique which provides an equal chance of selection. Confidentiality of the study was maintained by establishing codes instead of using names.

# Results

## Socio-demographic and economic characteristics of cases and controls households

This study was employed 300 study participants (100 cases and 200 controls) and the response rate was 100%. Of the study participants, 49(49.0%) of cases and 88(44.0%) of controls were males. The educational level of almost half 48(48.0%) of cases and two-third 130(65.0%) of the controls were illiterate. The average monthly household income was $55.60 USD (United States Dollars), of which 27(27.0%) of cases and 89(44.5%) of controls had less than $55.60 USD monthly household income. In both control and case households, more than half of the study participants 133(66.0%) of the controls and 63(63.0%) of the cases, rented government houses administered by *kebeles* (Table 1).

## The superstructure and privacy-related characteristics of the shared latrines among cases and controls

About two-thirds 62(62.0%) of the cases and 143(71.5%) of controls shared one latrines for 11 to 13 households, whereas 23(23.0%) of cases and 37(18.5%) of controls shared one latrine for 6 to 10 households. About one-third (30, 30%) case household latrines and one-fifth (43, 21.5%) of control household latrines superstructure were constructed with bricks or stones. About one-tenth 13(13.0%) of the cases households and more than half 107(53.7%) of controls shared latrines had no doors. Two-thirds 67(67.0%) of the cases and three-fourths 157(78.5%) of controls mentioned lack of privacy when using the latrines (Table 2).

## Shared latrine slab characteristics, latrine monitoring and collective decision-making practices

About 32(32.0%) of the case households latrines and nearly half of control households 99(49.5%) latrine had cracked or broken slabs. Almost two-thirds 121(60.5%) of the control households and half 53(53%) of the case households reported conflicts among latrine users. One fifth 26(26%) of case and a majority 166(83%) control households latrine were not monitored regularly by health extension workers. Most control households 174(87%), but only 31(31.0%) of the case households considered their latrines not clean (Table 3).

## Availability of water and hand-washing facilities in the shared latrines

Nearly one-third 31(31.0%) of control household had enough water available within or near the latrine for cleaning the sanitation facilities. A majority of the case 84(84.0%) and controls 165(82.5%) latrines had no hand washing facilities inside and/or near the latrine. Of those case households’ latrines that had handwashing facilities 16(16.0%) inside and/o near latrines, only 4 (25.0%) has water and 2(12.5%) water and soap (Table 4).

## Barriers associated with cleaning shared latrines in slums of Addis Ababa

This study identified six barriers significantly associated with not cleaning shared latrines in slums of District 05 in Lideta Sub-City, Addis Ababa. Households that shared a latrine and had a monthly income of less than $55.60 USD were 1.8 times (AOR = 1.8; 95% CI: 1.2-3.10) more likely to not clean the latrine than those that had a monthly income of $55.60 USD or above. Shared latrine users that felt a lack of privacy when using the latrines were 2.95 less likely to clean the latrine (AOR = 2.95; 95% CI: 1.60-5.43) than those who had a feeling of privacy. Users of shared latrines that had no locking latch were 4.6 times less likely to clean the latrine (AOR = 4.60; 95% CI: 2.43-8.79) compared to those users of public latrines that had a locking latch.

Households using shared latrines that were without adequate ventilation were 4.88 times (AOR: 4.88; 95% CI: 2.44-9.63) less likely to clean the latrine than those using shared latrine that had adequate ventilation. Users of shared latrines not monitored regularly by health extension workers were 2.86 times (AOR = 2.86; 95%CI: 1.32-6.21) less likely to clean the latrine than those that were monitored by health extension workers. Users who believed that there was not enough water at home for cleaning the latrine were 4.9 times (AOR = 4.91; 95% CI: 1.07-9.48) less likely to clean the latrine than those who believed there was enough water at home for cleaning the latrine (Table 5).

# Discussion

The main aim of this study was to determine the barriers to cleaning of shared latrines in slums of District 05, Lideta Sub-City of Addis Ababa, Ethiopia. To address this, an unmatched case-control study was employed. We found that the barriers to cleaning of shared latrines were monthly household income, users that felt a lack of privacy during latrine use, a shared latrine without locking latch, shared latrine without adequate ventilation, shared latrine not regularly monitored by health extension workers and a lack of enough water at home for cleaning the latrine.

Our findings showed the average household monthly income of less than $55.60 USD was inversely related to cleaning of shared latrines. This might be due to slum residents, who are commonly poor, give attention to their food security concern rather than the hygienic conditions of shared latrines. A previous study in slums of Addis Ababa found nearly 14.1% of the wealthiest households had access to improved latrine (not shared) compared to only 0.5% of the poorest who had unshared latrines [33]. Furthermore, due to lack of income, the affordability of latrine cleaning agents might be also challenge for slum dwellers. Low income status may hinders for purchasing latrine cleaning and handwashing agents [41].

Lack of privacy was one of the barriers to cleaning of the shared latrines, which might be attributed to them being built of poor construction materials that provided less privacy. A study in Nepal reported that lack of privacy for women to use latrine pushed to look for open defecation places [42]. Therefore, privacy is an important factor for latrine use and cleaning. Lack of privacy might be due to the poor cleanliness of the latrine. A study in Kisumu, Kenya that found that latrines constructed with iron sheets, which were likely to have a wooden slab, tended to be more dirty than facilities built of bricks with a cement floor [43]. The shared latrine without locking latch was also associated with not cleaning of latrine in our study is in contrast with a study in Tanzania revealed non-shared latrine had less likely to have lockable doors than shared latrines [44]. This might be due to presence of locking latch increase the sense of ownership of the shared latrine. If the shared latrine users had their own key, others will not use it which prevents to make the latrine clean.

A study in the Kibera slums in Nairobi found that respondents used shared latrines due to the scarcity of private household latrines and the poor condition of other sanitation facilities in that crowded area [45]. Consistent with our findings, Heijenen *et al*. reported that households sharing sanitation facilities were generally poorer than those that did not share, not necessarily because of sharing sanitation facilities but because of poverty [46]. A study in slums of Uganda showed that 68% of the shared latrine were observed to be significantly dirty [47]. Another study has revealed that shared sanitation facility users are not committed to cleaning shared latrines [48]. In our study, latrine door that had no locking latch was one of the barrier for latrine cleaning, which may exposed the latrine to a chance of everyone to use the latrine without specified users, in turn can compromise the hygiene of the latrine.

Our findings showed that not regularly monitoring of the latrine by health extension workers was a barriers for latrine cleaning, which might be due to the fact that when there is monitoring users are concerned about in implementing the health extension packages, water, sanitation and hygienic was one of the package. Other studies also showed that the presence of health extension program in Ethiopia changed the performance of health systems [49, 50]. The findings of our study and the previous studies in Ethiopia consistently showed the use of health extension program is immense and health extension workers consistent follow-up of latrines highly advisable.

This study also indicates that lack of water availability is one of the barriers to cleaning shared latrines. Unavailability of water not only latrine cleaning, but also causes diarrheal disease among children under five in slums [35]. For instance, in 2017 the WHO/UNICEF Joint Monitoring Program (JMP) report indicated that in 2015, 29.0% of the global population (2.1 billion people) lacked properly managed drinking water services and 61.0% (4.5 billion people) lacked adequate sanitation services [51], which compromises latrine cleaning practices. A study in Tanzania showed water scarcity was a barriers for national sanitation campaign [52].

This study used an unmatched case-control design and controlling of the confounders at the design stage was not possible. Further studies using matched case-control design may provide better evidence. The self-reported data may have bias by underestimate or overestimate the sanitation and hygiene status of the latrine [53], although most of the data was collected using observational checklist. A follow-up study design with a mixed method of data (quantitative and qualitative) may help to capture more of the practical situation.

# Conclusion

We found that the barriers for shared latrine cleaning were monthly household income, users that felt a lack of privacy during latrine use, shared latrines that had no locking latch, shared latrines without adequate ventilation, shared latrine not regularly monitored by health extension workers and lack of enough water at home for cleaning.

We recommended that the Addis Ababa Water and Sewerage Authority and other concerned bodies should implement the urban sanitation strategic plan in a well-organized and integrated manner by providing special attention to needs of the shared latrine users in slums of Addis Ababa. Promoting the cleaning of shared latrines through designing strategies for increasing the household monthly income by means of the urban productive safety net program, increasing the privacy of the latrines by fixing the superstructure, providing latrines with doors and locking latches, adjusting the latrines to allow good ventilation, regular monitoring of the latrines by health extension workers and by making enough water available for regular latrine cleaning. Previous findings also have showed that sanitation issues affecting all community members can be addressed in meetings that ensure collective decision making and formulation of rules for sanitation facilities [11].

# Acknowledgment

We thank GAMBY College of Medical and Business Sciences for the support we received from the inception to the finalization of the study. We highly acknowledge District 05 in Lideta Sub-City Health Office for the cooperation we received during data collection. We also thank study participants, data collectors and supervisors for their kind assistance during the study. We also highly appreciate Lisa Penttila for language editing of this manuscript.

# References

1.	UN-HABITAT. Urban features: children, slums first casualties. The millennium development goals and urban sustainability. Nairobi. United Nations Human Settlements Programme (UN-HABITAT)2006/7.

2.	UN-HABITAT. The Challenge of Slums. Global Report on Human Settlements: London and Sterling VA: Earth Scan Publications; 2003.

3.	Unger A. Children’s health in slum settings. Arch Dis Child. 2013;98:799–805.

4.	UN-HABITAT. State of the World's Cities 2010/11. Bridging the Urban Divide, Overview and Key Findings. United Nations Human Settlements Programme. Nairobi, Kenya2010/11.

5.	UN-HABITAT. Sustainable urbanizations: Local action for urban poverty reduction, emphasis on finance and planning. United Nations Human Settlement Program (UN-HABITAT), 21st Session of the Governing Council, Nairobi, Kenya2007.

6.	Ravaillon M, Chen S, Sangraula P. New evidence on the urbanization of global poverty. Washington DC: World Bank. 2007.

7.	UN-HABITAT. The state of African cities: Re-imagining sustainable urban transitions 2014.

8.	Shiras T, Cumming O, Brown J, Muneme B, Nala R, Dreibelbis R. Shared latrines in Maputo, Mozambique: exploring emotional well-being and psychosocial stress. BMC Int Health Hum Rights. 2018;18(30).

9.	Kwiringira J, Atekyereza P, Niwagaba C, Günther I. Descending the sanitation ladder in urban Uganda: evidence from Kampala Slums. BMC Public Health. 2014;14(624).

10.	Blackett I, Hawkins P, Heymans C. The missing link in sanitation service delivery: A review of fecal sludge management in 12 cities. Washington DC: WSP-World Bank research brief. 2014.

11.	Nelson KB, Karver J, Kullman C, Graham JP. User perceptions of shared sanitation among rural households in Indonesia and Bangladesh. PloS ONE. 2014;9(8):e103886.

12.	Mutisya M, Orindi B, Emina J, Zulu E, Ye Y. Is mortality among under-five children in Nairobi slums seasonal? Trop Med Int Health. 2010;15(1):132-9.

13.	Carlton E, Eisenberg J, Gold J, Cevallos W, Trostle J, Karen L. Heavy rainfall events and diarrhoea incidence: The role of social and environmental factors. Am J Epidemiol. 2013;179(3):344-52.

14.	Kwiringira J, Atekyereza P, Niwagaba C, Kabumbuli R, Rwabukwali C, Kulabako R, et al. Seasonal variations and shared latrine cleaning practices in the slums of Kampala city, Uganda. BMC Public Health. 2016;16(361)(361).

15.	Heijnen M, Cumming O, Peletz R, Chan G, Brown J, Baker K, et al. Shared sanitation versus individual household latrines: A systematic review of health outcomes. PLoS ONE. 2014;9(4):e93300(4).

16.	Heijnen M, Rosa G, Fuller J, Eisenberg J, Clasen T. The geographic and demographic scope of shared sanitation: An analysis of national survey data from low- and middle income countries. Trop Med Int Health. 2014;19(11):1334-45(11).

17.	Nelson K, Karver J, Kullman C, Graham J. User perceptions of shared sanitation among rural households in Indonesia and Bangladesh. PLoS ONE. 2014;9(8):e103886(8).

18.	Simiyu S, Swilling M, Cairncross S, Rheingans R. Determinants of quality of shared sanitation facilities in informal settlements: Case study of Kisumu, Kenya. BMC Public Health. 2017;17(68).

19.	Worldwatch-Institue. Our urban future. A Worldwatch Institue Report on progress towards a sustainable society. 24th edn ed. 24th ed. Washington, DC: The Worldwatch Institue. 2007.

20.	Bartlett S. Water sanitation and urban children: The need to go beyond improved provision. *Environ Urban*. 2003;15(2):57-70(2).

21.	Roba T. Assessment of the implementation of community-led total sanitation, hygiene, and associated factors in Diretiyara district, Eastern Ethiopia. PLoS ONE. 2017;12(4):e0175233(4).

22.	Collinet S, Babji S, Francis M, Kattula D, Premkumar P, Sarkar R, et al. Environmental factors associated with high fly densities and diarrhea in Vellore, India. *Appl Environ Microbiol*. 2015;81(17):6053-8(17).

23.	Strina A, Cairncross S, Barreto M, Larrea C, Prado M. Childhood diarrhea and observed hygiene behavior in Salvador, Brazil. Am J Epidemiol. 2003;157(11):1032-8(11).

24.	Alam Z, Rahman A, Al-Firoz A. Water supply and sanitation facilities in urban slums: A case study of Rajshahi City corporation slums. *Am J Civ Eng Archit*. 2013;1(1):1-6(1).

25.	Fadel M, Maroun R, Quba'a R, Mawla D, Sayess R, Massoud M, et al. Determinants of diarrhoea prevalence in urban slums: A comparative assessment towards enhanced environmental management. Environ Monit Assess. 2014;186(2):665-77(2).

26.	Owolabi A. Domestic water use, sanitation and diarrhea incidence among various communities of Ikare Akoko, Southwestern, Nigeria. Afr J Microbiol Res. 2012;6(14):3465-79(14).

27.	Irish S, Aiemjoy K, Torondel B, Abdelahi F, Ensink J. Characteristics of latrines in central Tanzania and their relation to fly catches. PLoS ONE. 2013;8(7):e67951(7).

28.	Nakagiri A, Kulabako R, Nyenje P, Tumuhairwe J, Niwagaba C, Kansiime F. Performance of pit latrines in urban poor areas: A case of Kampala, Uganda. Habitat Int. 2015;49:529-37.

29.	UN. The Sustainable Development Goals Report 2016. United Nations, New York2016.

30.	Shiras T, Cumming O, Brown J, Muneme B, Nala R, Dreibelbis R. Shared sanitation management and the role of social capital: Findings from an urban sanitation intervention in Maputo, Mozambique. Int J Env Res Public Health. 2018;15(10):2222.

31.	UN-HABITAT. Ethiopia: Addis Ababa Urban Profile. United Nation's Human Settlements Programme Regional and Technical Cooperation Division, Nairobi, Kenya2008.

32.	UN-HABITAT. The Ethiopian case of condominium housing. The integrated housing development program. United Nations Human Settlement Program (UN-HABITAT). Nairobi, Kenya2010.

33.	Beyene A, Hailu T, Faris K, Kloos H. Current state and trends of access to sanitation in Ethiopia and the need to revise indicators to monitor progress in the Post-2015 era. BMC Public Health. 2015;15(451).

34.	Adane M, Mengistie B, Kloos H, Medhin G, Mulat W. Sanitation facilities, hygienic conditions, and prevalence of acute diarrhea among under-five children in slums of Addis Ababa, Ethiopia: Baseline survey of a longitudinal study. PLoS ONE. 2017;12(8):e0182783.

35.	Adane M, Mengistie B, Medhin G, Kloos H, Mulat W. Piped water supply interruptions and acute diarrhea among under-five children in Addis Ababa slums, Ethiopia: A matched case-control study. PLoS ONE. 2017;12(7):e0181516.

36.	WHO, UNICEF. Progress on Drinking Water and Sanitation: Special Focus on Sanitation. World Health Organization and United Nations Children’s Fund Joint Monitoring Programme (JMP) for Water Supply and Sanitation. New York: Geneva2008.

37.	Schlesselman J, Stolley P. Case-control studies: Design, conduct, analysis. Oxford. Oxford University Press. 1982:144-70.

38.	Miettinen O. Individual matching with multiple controls in the case of all-or-none responses. Biometrics. 1969;25:339-55.

39.	Kuo C, Duan Y, Grady J. Unconditional or conditional logistic regression model for age-matched case–control data? Front Public Health. 2018;6(57).

40.	McNamee R. Confounding and confounders. Occup Environ Med. 2003;60(3):227-34.

41.	Hoque B. Handwashing practices and challenges in Bang-ladesh. Int J Env Health Res 2003;13(Suppl I):S81-S7.

42.	Bhatt N, Budhathoki SS, Lucero-Prisno DEI, Shrestha G, Bhattachan M, Thapa J, et al. What motivates open defecation? A qualitative study from a rural setting in Nepal. PLoS ONE. 2019;14(7):e0219246.

43.	Simiyu S, Swilling M, Cairncross S, Rheingans R. Determinants of quality of shared sanitation facilities in informal settlements: Case study of Kisumu, Kenya. BMC Public Health. 2017;17(68).

44.	Massa K, Kilamile F, Safari E, Seleman A, Mwakitalima A, Balengayabo JG, et al. Contributing to the debate on categorising shared sanitation facilities as ‘unimproved’: An account based on field researchers’ observations and householders’ opinions in three regions, Tanzania. PLoS ONE. 2017;12(11):e0185875.

45.	Simiyu S. Determinants of usage of communal sanitation facilities in informal settlements of Kisumu, Kenya. Environ Urban. 2016;28(1):241-58.

46.	Heijnen M, Routray P, Torondel B, Clasen T. Neighbour-shared versus communal latrines in urban slums: A cross-sectional study in Orissa, India exploring household demographics, accessibility, privacy, use and cleanliness. T Roy Soc Trop Med H. 2015;109(11):690-9.

47.	Tumwebaze IK, Niwagaba CB, Günther I, Mosler H-J. Determinants of households' cleaning intention for shared toilets: Case of 50 slums in Kampala, Uganda. Habitat international. 2014;41:108-13.

48.	Tumwebaze I, Mosler H. Shared toilet users collective cleaning and determinant factors in Kampala slums, Uganda. BMC Public Health. 2014;14(1260).

49.	Fetene N, Linnander E, Fekadu B, Alemu H, Omer H, Canavan M, et al. The Ethiopian health extension program and variation in health systems performance: What matters? PLoS ONE 2016;11(5):e0156438.

50.	Amouzou A, Kidanu A, Taddesse N, Silva R, Hazel E, Bryce J, et al. Using health extension workers for monitoring child mortality in real-time: Validation against household survey data in rural Ethiopia. PLos ONE. 2015;10(11):e0126909.

51.	WHO, UNCEF. WHO/UNICEF Joint Monitoring Programme 2017 report. 2017.

52.	Czerniewska A, Muangi WC, Aunger R, Massa K, Curtis V. Theory-driven formative research to inform the design of a national sanitation campaign in Tanzania. PLoS ONE 2019;14(8):e0221445.

53.	Vedachalam S, MacDonald LH, Shiferaw S, Seme A, Schwab KJ, investigators P. Underreporting of high-risk water and sanitation practices undermines progress on global targets. PLoS ONE. 2017;12(5):e0176272.

Table 1 Socio-demographic and economic characteristics among case and control households in slums of District 05, Lideta Sub-City, Addis Ababa

| **Variable** | **Category** | **Case (*N* = 100)** | **Control (*N* = 200)** | **COR (95%CI)** |
| --- | --- | --- | --- | --- |
|  |  | **n(%)** | **n(%)** |  |
| Age (years) | 25-34 | 23(23.0) | 52(26.0) | 0.7(0.4–1.4) |
|  | 35-44 | 34(34.0) | 73(36.5) | 0.8(0.5–1.4) |
|  | >44 | 43(43.0) | 75(37.5) | Ref |
| Sex | Male | 49(49.0) | 88(44.0) | 0.8(0.5–1.3) |
|  | Female | 51(51.0) | 112(56.0) | Ref |
| Marital status | Married | 76(76.0) | 152(76.0) | 1.6(0.8–3.5) |
|  | Single | 15(15.0) | 18(9.0) | 0.5(0.2–1.3) |
|  | Widowed | 5(5.0) | 21(10.5) | 0.9(0.3–3.0) |
|  | Divorced | 4(4.0) | 9(4.5) | Ref |
| Educational status | Illiterate | 48(48.0) | 130(65.0) | 0.3(0.1–1.1) |
|  | Read and write | 22(22.0) | 47(23.5) | 0.4(0.1-1.4) |
|  | Elementary | 24(24.0) | 18(9.0) | 1.1(0.3–4.2) |
|  | Secondary or above | 6(6.0) | 5(2.5) | Ref |
| Occupation | House wife | 44(44.0) | 90(45.0) | 0.9(0.4–2.0) |
|  | Government employee | 33(33.0) | 59(29.5) | 1.1(0.4–2.3) |
|  | Daily laborer | 12(12.0) | 31(15.5) | 0.7(0.3–1.9) |
|  | Merchant | 11(11.0) | 20(10.0) | Ref |
| Monthly household income ($US) | Less than $55.60 US | 27(27.0) | 89(44.5) | 2.2(1.3–3.6) |
|  | $55.60 US or more | 73(73.0) | 111(55.5) | Ref |
| Household size (persons) | 6 or more persons | 24(24.0) | 43(21.5) | 1.1(0.6–2.0) |
|  | 1-5 persons | 76(76.0) | 157(78.5) | Ref |
| House ownership | Rented from government (*kebele*) | 63(63.0) | 133(66.5) | 1.1(0.6-0.2) |
|  | Privately rented | 21(21.0) | 40(20.0) | 1.2(0.6–2.5) |
|  | Owned by householder | 16(16.0) | 27(13.5) | Ref |
| Number of rooms in house | ≤2 | 60(60.0) | 144(72.0) | 1.7(1.1–2.8) |
|  | >2 | 40(40.0) | 56(28.0) | Ref |

1, Reference category; *The average exchange rate for $1 USD was 20.0 birr from September to November 2017. COR (95%CI), Crude odds ratio at 95% confidence interval.

Table 2 Superstructure and privacy-related characteristics of shared latrines among case and control households in slums of District 05, Lideta Sub-City, Addis Ababa

|  |  | | **Case (*N* = 100)** | **Control (*N* = 200)** | **COR (95%CI)** |
| --- | --- | --- | --- | --- | --- |
| **Characteristics** | | **Category** | ***n*(%)** | ***n*(%)** |  |
| Number of households sharing latrine | | ≤5 | 15(15.0) | 20(10.0) | Ref |
|  |  | 6-10 | 23(23.0) | 37(18.5) | 0.8(0.4–1.9) |
|  |  | 11-13 | 62(62.0) | 143(71.5) | 0.6(0.3–1.2) |
| Superstructure materials of  shared latrine | | Bricks/stone | 30(30.0) | 43(21.5) | 1.1(0.60-2.1) |
|  |  | Mud/wood | 5(5.0) | 41(20.5) | 0.9(0.15-2.0) |
|  |  | Corrugated iron sheets | 65(65.0) | 121(58.0) | Ref |
| Adequate privacy during use of  shared latrine | | No | 33(33.0) | 157(78.5) | 7.4(4.3-12.6) |
|  |  | Yes | 67(67.0) | 43(21.5) | Ref |
| Shared latrine had a door | | No | 13(13.0) | 107(53.7) | 7.7(4.0-14.7) |
|  |  | Yes | 87(87.0) | 93(46.5) | Ref |
| Shared latrine had door with a locking latch | | No | 23(23.0) | 136(68.0) | 7.1(4.1-12.3) |
|  |  | Yes | 77(77.0) | 64(32.0) | Ref |
| Shared latrine had good ventilation | | No | 21(21.0) | 143(71.5) | 9.4(5.3-16.7) |
|  |  | Yes | 79(79.0) | 57(28.5) | Ref |

Table 3 Latrine slab, monitoring practices and other characteristics of shared latrines among case and control households in slums of District 05, Lideta Sub-City, Addis Ababa

| **Characteristics** | **Category** | **Case (*N* = 100)** | **Control (*N* = 200)** | **COR (95%CI)** |
| --- | --- | --- | --- | --- |
|  |  | **n(%)** | **n(%)** |  |
| Cracked or broken slab | No | 68(68.0) | 101(50.5) | 2.1(1.3-3.4) |
|  | Yes | 32(32.0) | 99(49.5) | Ref |
| The latrine pit was full | No | 75(75.0) | 129(64.5) | 0.6(0.3-1.0) |
|  | Yes | 25(25.0) | 71(35.5) | Ref |
| Users participated collectively in decision making | No | 23(23.0) | 147(73.5) | 9.3(5.3-16.3) |
|  | Yes | 77(77.0) | 53(26.5) | Ref |
| Users experienced conflict | Yes | 53(53.0) | 121(60.5) | 1.4(0.8-2.2) |
|  | No | 47(47.0) | 79(39.5) | Ref |
| Regular monitoring of the latrines by health extension workers | No | 26(26.0) | 166(83.0) | 13.9(7.8-24.8) |
|  | Yes | 74(74.0) | 34(17.0) | Ref |
| Latrine was considered to be clean by users | Yes | 69(69.0) | 26(13.0) | 14.9(8.2-26.9) |
|  | No | 31(31.0) | 174(87.0) | Ref |

Ref, Reference category

Table 4 Availability of water and hand washing facilities in shared latrines among case and control households in slums of District 05 in Lideta Sub-City, Addis Ababa

| **Variables** | **Category** | **Case (*N* = 100)** | **Control (*N* = 200)** | **COR (95%CI)** |
| --- | --- | --- | --- | --- |
|  |  | **n(%)** | **n(%)** |  |
| Enough water was available at home for cleaning the latrine^¥^ | No | 69(69.0) | 194(97.0) | 0.7(0.8-6.3) |
|  | Yes | 31(31.0) | 6(3.0) | Ref |
| Hand washing facilities inside and/or near the latrine* | No | 84(84.0) | 165(82.5) | 1.1(0.7-2.5) |
|  | Yes | 16(16.0) | 35(17.5) | Ref |
| Presence of water in the hand washing facilities* | No | 12(75.0) | 27(77.1) | 0.8(0.9-1.7) |
|  | Yes | 4(25.0) | 8(22.9) | Ref |
| Availability of soap near the hand washing facilities* | No | 14(87.5) | 30(85.7) | 1.16(0.8-1.2) |
|  | Yes | 2(12.5) | 5(14.3) | Ref |

Ref, Reference category

*Not included during the logistic regression analysis due to the presence of zero frequency either in the case or control households.

^¥^The quantity of the available water was not measured and only the perception that they have enough water at home for latrine cleaning was studied.

Table 5 Barriers independently associated with cleaning of shared latrines in slums of District 05, Lideta Sub-City, Addis Ababa

| **Variables** | **Category** | **Cases (N = 100)** | **Controls (N = 200)** | **AOR (95%CI)** |
| --- | --- | --- | --- | --- |
|  |  | **n(%)** | **n(%)** |  |
| Household monthly income (USD) | Less than $55.60 | 27(27.0) | 89(44.5) | 1.8(1.2-3.10) |
|  | $55.60 or more | 73(73.0) | 111(55.5) | Ref |
| Feeling of privacy when using latrine | No | 33(33.0) | 157(78.5) | 2.95(1.60-5.43) |
|  | Yes | 67(67.0) | 43(21.5) | Ref |
| Latrine had a locking latch | No | 23(23.0) | 136(68.0) | 4.60(2.43-8.79) |
|  | Yes | 77(77.0) | 64(32.0) | Ref |
| Latrine had adequate ventilation | No | 21(21.0) | 143(71.5) | 4.88(2.44-9.63) |
|  | Yes | 79(79.0) | 57(28.5) | Ref |
| Regular monitoring of the latrine by health extension workers^¥^ | No | 26(26.0) | 166(83.0) | 2.86(1.32-6.21) |
|  | Yes | 74(74.0) | 34(17.0) | Ref |
| Enough water was available at home for cleaning the latrine* | No | 69(69.0) | 194(97.0) | 4.91(1.07-9.48) |
|  | Yes | 31(31.0) | 6(3.0) | Ref |

Ref, Reference category

^¥^ Monitoring by health extension workers (HEWs) were done by regular visit to the latrine and feedback provided to users about keeping the latrine clean.
